# Influence of tirofiban on stroke outcome after mechanical thrombectomy in acute vertebrobasilar artery occlusion

**DOI:** 10.1186/s12883-022-02996-5

**Published:** 2022-12-09

**Authors:** Xiding Pan, Mengyi Xu, Yuxiang Fei, Shiteng Lin, Yapeng Lin, Jianjun Zou, Jie Yang

**Affiliations:** 1 Department of Pharmacy, Nanjing First Hospital, Nanjing Medical University, 68 Changle Road, Nanjing, China; 2 Department of Pharmacy, Nanjing First Hospital, China Pharmaceutical University, Nanjing, China; 3 Department of Neurology, Nanjing First Hospital, Nanjing Medical University, Nanjing, China; 4 School of Basic Medicine and Clinical Pharmacy, China Pharmaceutical University, Nanjing, China; 5 International Clinical Research Center & Department of Neurology, Clinical Medical College and The First Affiliated Hospital of Chengdu Medical College, Chengdu, China; 6 Department of Neurology, Sichuan Provincial People’s Hospital, University of Electronic Science and Technology of China, 32 Second Section of Yihuanxi Road, Chengdu, China

**Keywords:** Vertebrobasilar artery occlusion, Antiplatelet, Tirofiban, Mechanical thrombectomy

## Abstract

**Background:**

Even undergoing mechanical thrombectomy (MT), patients with acute vertebrobasilar artery occlusion (AVBAO) still have a high rate of mortality. Tirofiban is a novel antiplatelet agent which is now widely empirically used in acute ischemic stroke (AIS). In this study, we aimed to evaluate the safety and efficacy of tirofiban as adjunctive therapy for MT in AVBAO.

**Methods:**

From October 2016 to July 2021, consecutive AVBAO patients receiving MT were included in the prospective stroke registry. The short-term outcomes were (1) symptomatic intracerebral hemorrhage (sICH); (2) in-hospital death; (3) National Institute of Health Stroke Scale (NIHSS) at discharge. The Long-term outcomes were: (1) modified Rankin Scale (mRS) at 3 months; (2) death at 3 months.

**Results:**

A total of 130 eligible patients were included in the study, 64 (49.2%) patients received tirofiban. In multivariate regression analysis, no significant differences were observed in all outcomes between the tirofiban and non-tirofiban group [sICH (adjusted OR 0.96; 95% CI, 0.12–7.82, *p* = 0.97), in-hospital death (adjusted OR 0.57; 95% CI, 0.17–1.89, *p* = 0.36), NIHSS at discharge (95% CI, -2.14–8.63, *p* = 0.24), mRS (adjusted OR 1.20; 95% CI, 0.40–3.62, *p* = 0.75), and death at 3 months (adjusted OR 0.83; 95% CI, 0.24–2.90, *p* = 0.77)].

**Conclusions:**

In AVBAO, tirofiban adjunctive to MT was not associated with an increased risk of sICH. Short-term (in-hospital death, NIHSS at discharge) and long-term outcomes (mRS and death at 3 months) seem not to be influenced by tirofiban use.

## Background

Acute vertebrobasilar artery occlusion (AVBAO) leads to a high rate of mortality and disability. It accounts for approximately 1% of all ischemic strokes [1, 2]. Although Mechanical thrombectomy (MT) has been applied to acute anterior circulation stroke (ACS) for more than 7 years, the evidence of MT in AVBAO was still limited. Two previously published randomized controlled trials (RCT), the BEST study and the BASICS study, both failed to prove the superiority of MT over standard medical treatment[3, 4]. However, just recently, two RCTs (BAOCHE study and ATTENTION study) which focused on moderate-to-severe stroke (NIHSS ≥ 10), first confirmed that MT significantly improved the functional outcome of AVBAO[5, 6]. Patients with AVBAO often have typical vascular risk factors, one of the most common stroke subtypes is large artery atherosclerosis (LAA)[7–9]. During the MT procedure, endothelial injuries may lead to platelet activation which may cause in situ thrombosis [10]. Thus, the use of antiplatelet agents during the perioperative period may improve MT outcomes. Tirofiban is a non-peptide, selective, and reversible antiplatelet agent. It inhibits the final pathway of platelet aggregation. Previous studies have shown that tirofiban could improve the clinical outcome of anterior circulation occlusion (ACO) after MT. However, for posterior circulation stroke, the efficacy evidence of tirofiban is limited. In this study, we aimed to evaluate the safety and efficacy of tirofiban as adjunctive therapy for MT in AVBAO.

## Methods

### Study design and patient enrolment

From October 2016 to July 2021, all acute stroke patients with basilar and vertebral artery occlusion receiving MT were enrolled from the Nanjing First Hospital and The First Affiliated Hospital of Chengdu Medical College. The protocol of the Stroke Registry was approved by the hospital ethics committee and informed consent was obtained. The patient’s eligibility for MT was evaluated by neuro-interventionist. All methods were performed in accordance with the relevant guidelines and regulations [11].

Tirofiban was considered for application in the following conditions: (1) severe residual stenosis (≥ 50%) after thrombectomy; (2) rescue treatment with stenting or balloon angioplasty or failed thrombectomy; (3) thrombectomy attempts ≥ 3 times; (4) severe atherosclerosis with a high risk of reocclusion. After 0.25–1 mg intra-arterial bolus, tirofiban was continuously intravenously administered for 16–24 h at a rate of 0.1–0.15 μg/kg/min after MT.

### Data collection and outcome measures

Baseline characteristics were collected on admission. Computerized tomography (CT) or magnetic resonance imaging (MRI) was performed before and 24 h after MT to exclude intracranial bleeding. Telephone follow-up was performed to evaluate long-term outcomes at 3 months after stroke.

### Outcome measures

Short-term outcomes were: (1) symptomatic intracerebral hemorrhage (sICH), according to the ECASS II classification [12]; (2) in-hospital death; (3) National Institute of Health Stroke Scale (NIHSS) at discharge.

Long-term outcomes were: (1) modified Rankin Scale (mRS) at 3 months (good outcome = mRS score 0–2); (2) death at 3 months.

### Statistical analysis

Baseline characteristics were presented as mean (Standard Deviation); median (interquartile range) or n (%). Mann–Whitney U-test, independent sample *t* test or Fisher exact test were used to compare the outcomes of the two groups. The influence of tirofiban on each outcome was evaluated by the multivariate logistic regression model. Confounding factors were identified as clinically relevant and with *p* < 0.1 on univariate analysis. Statistical analyses were performed by SPSS 22.0 (IBM Inc., Armonk, NY, USA). A statistically significant difference was defined as *p* < 0.05.

## Results

From October 2016 to July 2021, a total of 130 eligible patients were included in the study. Of them, 64 (49.2%) patients received tirofiban. Tirofiban group had a higher rate of large artery atherosclerosis (73% in tirofiban group vs. 53% in non-tirofiban group) and the non-tirofiban group had a higher rate of cardioembolism (38% in non-tirofiban group vs. 17% in tirofiban group). This discrepancy may be the origin of the differences in baseline characteristics that the tirofiban group had a higher rate of hypertension (*p* = 0.012), a higher level of LDL (*p* = 0.011), and non-tirofiban group had a higher rate of atrial fibrillation (*p* = 0.018), and a higher level of PT/INR (*p* < 0.001) (maybe caused by warfarin use to treat atrial fibrillation). The baseline characteristics of included patients were presented in Table 1.

**Table 1:** Baseline characteristics of included patients^a^

	**Tirofiban** ***n* = 64**	**Without Tirofiban** ***n* = 66**	***p* Value**
Age, y	65.8 ± 11.9	67.1 ± 13.0	0.069
Male	50 (78.1)	46 (69.7)	0.321
Hypertension	50 (78.1)	38(57.6)	0.012
Diabetes	17 (26.6)	10 (15.2)	0.109
Hyperlipidemia	3 (4.7)	2 (3.0)	0.678
Atrial fibrillation	7 (10.9)	18 (27.3)	0.018
Coronary artery disease	6 (9.4)	9 (13.6)	0.447
Previous TIA/stroke	15 (23.4)	16 (34.8)	0.192
NIHSS on admission	16 (8–32)	21 (12–28)	0.431
OTT, min	314 (195–436)	240 (155–405)	0.238
SBP	143 ± 23	145 ± 27	0.553
PT/INR	0.95 (0.91–1.00)	1.00 (0.95–1.06)	< 0.001
LDL	3.1 (2.5–3.6)	2.7 (2.1–3.1)	0.011
Glucose	6.6 (5.7–8.5)	6.7 (5.2–7.8)	0.566
Creatinine	71 (57–87)	67 (56–100)	0.680
TOAST classification			
Large artery atherosclerosis	47 (73.4)	35 (53.0)	0.016
Cardioembolism	11 (17.2)	25 (37.9)	0.008
Other	6 (9.4)	6 (9.1)	0.955
Occlusion site			
Basilar artery	28 (43.8)	37 (56.1)	0.160
Vertebral artery	36 (56.3)	5 (7.6)	< 0.001
TICI ≥ 2b	54 (84.4)	40 (74.1)	0.166
Previous IV tPA	16 (25.0)	26 (39.4)	0.079
Premorbid mRS(0–2)	8 (12.3)	8 (12.6)	0.919

*TIA* transient ischemic attack, *NIHSS* National Institutes of Health Stroke Scale, *OTT* onset to treatment time, *SBP* systolic blood pressure, *PT/INR* prothrombin time and international normalized ratio, *LDL* low density lipoprotein, *TICI* Thrombolysis in Cerebral Infarction grade, *tPA* tissue plasminogen activator

^a^Variables were expressed as mean ± SD or median (IQR) for continuous variables; n (%) for categorical variables

### Short-term outcomes

During hospitalization, tirofiban group compared with the control, sICH (4.7% vs. 12.1%; *p* = 0.128), NIHSS score at discharge (10 vs.13; *p* = 0.352) was not significantly different between the two groups. However, in-hospital death (14.1% vs. 28.8%; *p* = 0.041) was significantly lower in the tirofiban group. The results of short-term outcomes are summarized in Table 2.

**Table 2:** Univariate analysis of efficacy and safety outcomes

Outcomes	Tirofiban *n* = 64	Without Tirofiban *n* = 66	*p* Value
sICH	3 (4.7)	8 (12.1)	0.128
**In-hospital death**	**9 (14.1)**	**19 (28.8)**	**0.041**
NIHSS at discharge	10 (2–34)	13 (2–42)	0.352
mRS 0–2, 3 months^a^	29 (46.0)	25 (38.5)	0.386
**Death at 3 months**^**a**^	**16 (25.4)**	**30 (46.2)**	**0.014**

*sICH* symptomatic intracerebral hemorrhage, *NIHSS* National Institutes of Health Stroke Scale, *mRS* modified Rankin Scale

^a^2 patients were lost to follow-up

### Long-term outcomes

AT 3 months, 29 (46.0%) patients in tirofiban group and 25 (38.5%) in the control group had favorable outcomes, which were not significantly different (*p* = 0.386). 16 (25.4%) patients in tirofiban group were dead at 3 months, which was lower than control group (46.2%) (*p* = 0.014) (see Table 2).

### Multivariate regression analysis

We used multivariable logistic regression analysis to adjust the potential confounders. The results showed that the use of tirofiban was not associated with sICH (adjusted OR 0.96; 95% CI, 0.12–7.82, *p* = 0.97), in-hospital death (adjusted OR 0.57; 95% CI, 0.17–1.89, *p* = 0.36), NIHSS at discharge (95% CI, -2.14–8.63, *p* = 0.24). At 3 months, favorable outcome (adjusted OR 1.20; 95% CI, 0.40–3.62, *p* = 0.75) and death rate (adjusted OR 0.83; 95% CI, 0.24–2.90, *p* = 0.77) were not influenced by tirofiban either (see Table 3).

**Table 3:** Multivariate regression analysis to determine the influence of tirofiban on safety and efficacy outcomes

Outcomes	OR	95% CI	Adjusted *p* Value
sICH^a^	0.96	0.12 – 7.82	0.97
In-hospital death^b^	0.57	0.17 – 1.89	0.36
NIHSS at discharge^c^	–	-2.14 – 8.63	0.24
mRS 0–2, 3 months^d^	1.20	0.40 – 3.62	0.75
Death at 3 months^e^	0.83	0.24 – 2.90	0.77

*OR* odds ratio, *CI* confidence interval, *sICH* symptomatic intracerebral hemorrhage, *NIHSS* National Institutes of Health Stroke Scale, *mRS* modified Rankin Scale

^a^Adjusted for age, NIHSS on admission, TOAST classification, atrial fibrillation, PT/INR, creatinine

^b^Adjusted for age, NIHSS on admission, TOAST classification, PT/INR, TICI ≥ 2b

^c^Adjusted for age, NIHSS on admission, TOAST classification, previous TIA/stroke, creatinine, TICI ≥ 2b

^d^Adjusted for age, NIHSS on admission, TOAST classification, previous TIA/stroke, creatinine, TICI ≥ 2b

^e^Adjusted for age, NIHSS on admission, TOAST classification, coronary artery disease, previous TIA/stroke, creatinine, TICI ≥ 2b

### Sensitivity analysis

LAA patients were further analyzed. The definition of LAA was in accordance with the Trial of ORG 10,172 in Acute Stroke Treatment (TOAST) criteria [13]. Multivariable logistic regression analysis was performed in two models. Model 1 was adjusted by the same confounders of overall AVBAO patients. Model 2 was adjusted by confounders that showed *p* < 0.1 on univariate analysis in LAA patients. The results revealed that in-hospital death, death at 3 months, and the favorable outcome were still unaffected by tirofiban (see Table 4).

**Table 4:** Efficacy and safety outcomes of LAA subgroup

**Outcomes**	**Tirofiban** ***n* = 47**	**Without Tiofiban** ***n* = 35**	***p* Value**		**Mode l**			**Mode 2**	
				**OR**	**95% CI**	**Adjusted** ***p* Value**	**OR**	**95% CI**	**Adjusted** ***p* Value**
**In-hospital death**	7 (14.9)	11 (31.4)	0.074	0.691	0.197–2.425	0.564 ^b^	0.691	0.197–2.425	0.564 ^e^
**Death at 3 months**^a^	12 (26.1)	16 (45.7)	0.066	0.779	0.192–3.164	0.727 ^c^	0.989	0.254–3.855	0.988 ^f^
**mRS 0–2, 3 months**^a^	21 (45.7)	14 (40.0)	0.611	0.836	0.268–2.613	0.759 ^d^	0.964	0.328–2.836	0.947 g

*LAA* large artery atherosclerosis, *OR* odds ratio, *CI* confidence interval, *mRS* modified Rankin Scale

^a^1 patient were lost to follow-up

^b^Adjusted for age, PT/INR, TICI ≥ 2b

^c^Adjusted for age, coronary artery disease, previous TIA/stroke, creatinine, TICI ≥ 2b

^d^Adjusted for age, previous TIA/stroke, creatinine, TICI ≥ 2b

^e^Adjusted for age, PT/INR, TICI ≥ 2b

^f^Adjusted for age, previous TIA/stroke, PT/INR

^g^Adjusted for age, previous TIA/stroke

## Discussion

Acute vertebrobasilar artery occlusion (AVBAO) leads to high mortality and disability. In the MT procedure, a high recanalization rate (81–92%) was achieved in AVBAO patients [14, 15]. However, only about 30% of them obtained good functional outcomes [3, 9], which is lower than patients with anterior circulation occlusion (ACO) (approximately 50%) [16, 17]. The association between successful recanalization and good functional outcome was still controversial [18, 19].

In AVBAO, large artery atherosclerosis is one of the main subtypes of stroke[7–9]. A previous observational study showed that MT treatment might be more effective for LAA patients than for cardioembolism (CE) patients [20]. Thrombus histology analysis showed that vertebrobasilar artery thrombus contains a higher amount of red blood cells than anterior circulation [21]. These results indicated that thrombus growth in situ may be a major cause of thrombogenesis in the posterior circulation. Moreover, due to different collateral circulation patterns and lesser infarct volume, the hemorrhagic transformation was less likely to occur in AVBAO than in ACO [22]. For the reasons mentioned above, we presumed that AVBAO patients may benefit from adjunctive antiplatelet therapy in MT.

Tirofiban is a low-molecular-weight nonpeptide agent with a plasma half-life is about 2 h. It has a rapid onset and short duration of action, which makes the platelet function reversible within 4 h of cessation of infusion. Tirofiban is approved by The Food and Drug Administration and indicated for patients undergoing percutaneous coronary interventions (PCI) in the setting of ischemic heart disease. However, currently, it is extensively used off-label as adjunctive antiplatelet agent in selected patients with acute ischemic stroke (AIS) undergoing mechanical thrombectomy (MT). Such off-label use may prevent thrombosis events caused by the MT procedure and increase the recanalization rate. However, it has potential safety, ethical, and legal issues. In AIS, the optimum dosage of tirofiban and the characteristics of potential beneficiaries are not yet well-established. Moreover, although the overall incidence is low, adverse effects such as bleeding and thrombocytopenia may be life-threatening [23].

Several studies have been conducted to evaluate the efficacy and safety of tirofiban in MT for ACO [24–30]. All of them were observational studies and some of them reported conflicting findings. A subsequent meta-analysis showed that for stroke patients (most of them were ACO), tirofiban combined with MT improved 3 months functional outcome, reduced the risk of 3 months death, and did not increase the risk of sICH [30]. As for AVBAO, the efficacy and safety evidence of tirofiban is limited.

So far, only one study published by Sun et al. [31] aimed to evaluate tirofiban use in posterior circulation infarction. Their results revealed that the risk of sICH, 3 months mortality, and functional outcomes did not differ between the tirofiban and non-tirofiban groups. In our study, we reached the same conclusions. However, there were some differences between Sun et al. and our study. Firstly, Sun et al. only included atherosclerosis patients, but our study included all stroke types. Secondly, we found that NIHSS at discharge also had no significant statistical difference between the two groups. Further subgroup analysis showed that tirofiban was not associated with short-term and long-term outcomes in LAA subgroup patients either.

In conclusion, our study failed to prove the efficacy of tirofiban in AVBAO patients who underwent MT. This finding was inconsistent with the results in ACO patients, which showed that tirofiban improved 3 months functional outcome and reduced the risk of 3 months death [30]. These discrepancies may be attributed to several reasons. Firstly, patients with AVBAO were much more severe than ACO (median NIHSS 32 *vs*. 17) [3, 17]. The antithrombotic agent has classically been used for mild to moderate stroke [32]. The BASICS study suggested that when the antithrombotic agent was used without another more aggressive therapy (intravenous thrombolysis or mechanical thrombectomy), patients with AVBAO would not have good outcomes [33]. Secondly, the clinical outcomes of MT in AVBAO were mainly correlated with stroke severity and the success of the operation (such as admission NIHSS score, Alberta Stroke Program Early CT score, thrombolysis in cerebral infarction score, and residual stenosis after the procedure) [34–36]. The traditional prognostic factors such as gender, glucose, blood pressure, and comorbidities were not crucial in AVBAO [37–39]. Thus, the clinical outcomes of AVBAO may not be influenced by adjunctive antiplatelet therapy. Our results indicated that improving interventional techniques to reach more effective recanalization appears to be more critical for AVBAO patients.

Our study has several limitations. First, it is a retrospective analysis of a prospective observational study, and the number of patients is limited. Thus, the results should be interpreted with caution. Second, although the patient selection criteria and the dosage range of tirofiban was in accordance with practice guideline, the specific dosage varied for individual use, and it was mainly determined by clinician’s personal assessment. This may bias some effects on safety and efficacy outcomes. Third, some important factors such as prior antithrombotic agents use which may influence the sICH rate, were not recorded in the study. In this study, all patients in the study were enrolled in China, the findings of our study may not be applicable to global populations.

## Conclusions

In summary, in AVBAO, tirofiban as adjunctive therapy to MT was not associated with an increased risk of sICH. Short-term and long-term outcomes seemed not to be influenced by tirofiban use.

## Data Availability

Data are available on request from the corresponding author.

## Abbreviations

AVBAO: Acute vertebrobasilar artery occlusion
MT: Mechanical thrombectomy
CT: Computerized tomography
ACO: Anterior circulation occlusion
MRI: Magnetic resonance imaging
sICH: Symptomatic intracerebral hemorrhage
NIHSS: National Institute of Health Stroke Scale
mRS: Modified Rankin Scale
LAA: Large artery atherosclerosis
CE: Cardio embolism
PCI: Percutaneous coronary interventions
AIS: Acute ischemic stroke
