# Correction: Influence of tirofiban on stroke outcome after mechanical thrombectomy in acute vertebrobasilar artery occlusion

**DOI:** 10.1186/s12883-022-03043-z

**Published:** 2022-12-28

**Authors:** Xiding Pan, Mengyi Xu, Yuxiang Fei, Shiteng Lin, Yapeng Lin, Jianjun Zou, Jie Yang

**Affiliations:** 1 Department of Pharmacy, Nanjing First Hospital, Nanjing Medical University, 68 Changle Road, Nanjing, China; 2 Department of Pharmacy, Nanjing First Hospital, China Pharmaceutical University, Nanjing, China; 3 Department of Neurology, Nanjing First Hospital, Nanjing Medical University, Nanjing, China; 4 School of Basic Medicine and Clinical Pharmacy, China Pharmaceutical University, Nanjing, China; 5 International Clinical Research Center & Department of Neurology, Clinical Medical College and The First Affiliated Hospital of Chengdu Medical College, Chengdu, China; 6 Department of Neurology, Sichuan Provincial People’s Hospital, University of Electronic Science and Technology of China, 32 Second Section of Yihuanxi Road, Chengdu, China

**Correction: BMC Neurol 22, 460 (2022)**

**https://doi.org/10.1186/s12883-022-02996-5**

Following publication of the original article [1], the authors reported an error to author Jie Yang’s affiliation. Affiliation 5 was removed for author Jie Yang.

The original article [1] has been updated.
